# Long-read sequencing of the zebrafish genome reorganizes genomic architecture

**DOI:** 10.1186/s12864-022-08349-3

**Published:** 2022-02-10

**Authors:** Yelena Chernyavskaya, Xiaofei Zhang, Jinze Liu, Jessica Blackburn

**Affiliations:** 1 Department of Cellular & Molecular Biochemistry, University of Kentucky, Lexington, KY 40536 USA; 2 Markey Cancer Center at the University of Kentucky, Lexington, KY 40536 USA; 3 Department of Computer Science, University of Kentucky, Lexington, KY 40536 USA; 4 Department of Biostatistics, Virginia Commonwealth University, Richmond, USA

**Keywords:** Nanopore, MinION, *Danio rerio*, Reference assembly, Transposon

## Abstract

**Background:**

Nanopore sequencing technology has revolutionized the field of genome biology with its ability to generate extra-long reads that can resolve regions of the genome that were previously inaccessible to short-read sequencing platforms. Over 50% of the zebrafish genome consists of difficult to map, highly repetitive, low complexity elements that pose inherent problems for short-read sequencers and assemblers.

**Results:**

We used long-read nanopore sequencing to generate a de novo assembly of the zebrafish genome and compared our assembly to the current reference genome, GRCz11. The new assembly identified 1697 novel insertions and deletions over one kilobase in length and placed 106 previously unlocalized scaffolds. We also discovered additional sites of retrotransposon integration previously unreported in GRCz11 and observed the expression of these transposable elements in adult zebrafish under physiologic conditions, implying they have active mobility in the zebrafish genome and contribute to the ever-changing genomic landscape.

**Conclusions:**

We used nanopore sequencing to improve upon and resolve the issues plaguing the current zebrafish reference assembly, GRCz11. Zebrafish is a prominent model of human disease, and our corrected assembly will be useful for studies relying on interspecies comparisons and precise linkage of genetic events to disease phenotypes.

**Supplementary Information:**

The online version contains supplementary material available at 10.1186/s12864-022-08349-3.

## Background

A high-quality reference genome strengthens the relevance of model organisms to their human counterparts. Complete genomic data allows for the accurate evaluation of gene regulation, identification of mutations in disease states, assessment of evolutionarily conserved functional elements, and most importantly, permits manipulation of genetic sequence to create valuable tools to study human diseases. However, most reference genomes contain regions of poor coverage, or gaps, in the genome assembly. These gaps can be kilobases in length; next-generation sequencing (NGS), which produces short reads of 300 base pairs or less, cannot resolve these issues [4]. Consequently, long-read sequencing technologies, such as Pacific Biosystems (PacBio) and Oxford nanopore sequencing, have emerged as a means to generate reads that extend beyond 100 kilobase pairs (Kbp). These long read lengths can span across areas of poor coverage to fill the gaps in the genomic sequence.

Researchers have used the zebrafish to study embryonic development since the 1960s [3, 40], but its emergence as a model of human disease has dictated the need for an accurate genomic assembly. Over 70% of genes associated with disease states in humans have a direct functional ortholog in zebrafish. A comparative map of the zebrafish genome relative to human has been generated for the express purpose of identifying such orthologs [44]. Researchers can engineer zebrafish models of human disease using these genetic references by perturbing the counterpart orthologous genes [15, 31]. Therefore, having a quality reference genome is indispensable for molecular genetics in the zebrafish system.

However, several factors of the zebrafish genome complicate current assembly methods. First, the teleost genome has undergone multiple genome duplications, the most recent of which occurred after the divergence of the ray- and lobe-finned fishes more than 300 million years ago [1]. Duplicate genes may exhibit redundancy, dosage dependency, or other functions that are difficult to predict [1, 12, 34]. Additionally, many duplicate regions exist on different chromosomes from one another or in a state where their identification, annotation, and mapping are difficult due to increased sequence divergence or existence on unlocalized contigs [34]. The second obstacle to assembling a high-quality reference for zebrafish is the excessive repeat regions present in the *Danio rerio* genome. A comprehensive study by Chopin et al. compared 23 vertebrate genomes, including zebrafish, human, and mouse, and found that transposable elements (TEs) and repeats comprised more than 50% of the entire zebrafish genome, which is more than any other species examined [7]. These repeats can extend several megabase pairs and pose a formidable challenge to the accurate assembly of the zebrafish genome. Sequencing-by-synthesis technologies like NGS cannot generate reads long enough to span these regions.

Recently, researchers used nanopore sequencing to assess the mobility of a six kilobase transposable LINE-1 element in the human genome relative [13]. Old TEs accumulate enough sequence diversity over time to be distinct. However, young, mobile TEs are identical to their source element, making it impossible for short-read sequencing to resolve each TE [22]. Nanopore sequencing overcomes the size constraints imposed by NGS since native genomic DNA of any length can be fed through and “read” by each nanopore without the need for synthesis reactions [17]. Nanopore sequencing allows for sequencing across repeat regions such as telomeres, centromeres, and TEs [13, 16, 27]. However, a lower base-pair read accuracy somewhat offsets the benefit of extended read length, so most assemblies generated with long-read sequencing use supplementary short-read sequencing or increased depth to overcome this issue [27, 36].

According to the Genome Reference Consortium, the current zebrafish reference genome (GRCz11) contains 1448 unresolved gaps across all 25 chromosomes and 967 extrachromosomal unplaced contigs. Many of these regions are large enough to contain genes. However, because they lack concrete chromosomal locations, their regulation remains a mystery since it is impossible to know which cis- (promoters) or trans- (enhancers) acting elements govern their expression. Additionally, these statistics apply only to known issues with the current assembly and do not include errors that have yet to be defined. Recently, several nanopore-based zebrafish genomes have been deposited into the genome repository; however, their data have not been published. Without assessment or analysis of novel features or discoveries, these assemblies remain limited in utility and accessibility to the general scientific community. Additionally, the pipelines used to construct them remain largely unknown, hindering future improvements. We report a complete, de novo hybrid assembly of the zebrafish genome using nanopore long-read sequencing and NGS short-reads and an assessment of several assembly pipelines. We compared our assembly to the current zebrafish reference genome assembly, GRCz11, to resolve the placement of formally unlocalized contigs and identify new sequence indels. We also discovered novel retrotransposon insertion sites previously unreported in the reference assembly that contributes to genetic heterogeneity between different zebrafish model strains. These findings demonstrate the nanopore sequencing platform’s ease and universal application in resolving difficult to map regions and genomic gaps.

## Results

### Long-reads sequence across complex genomic regions

**Fig. 1:**
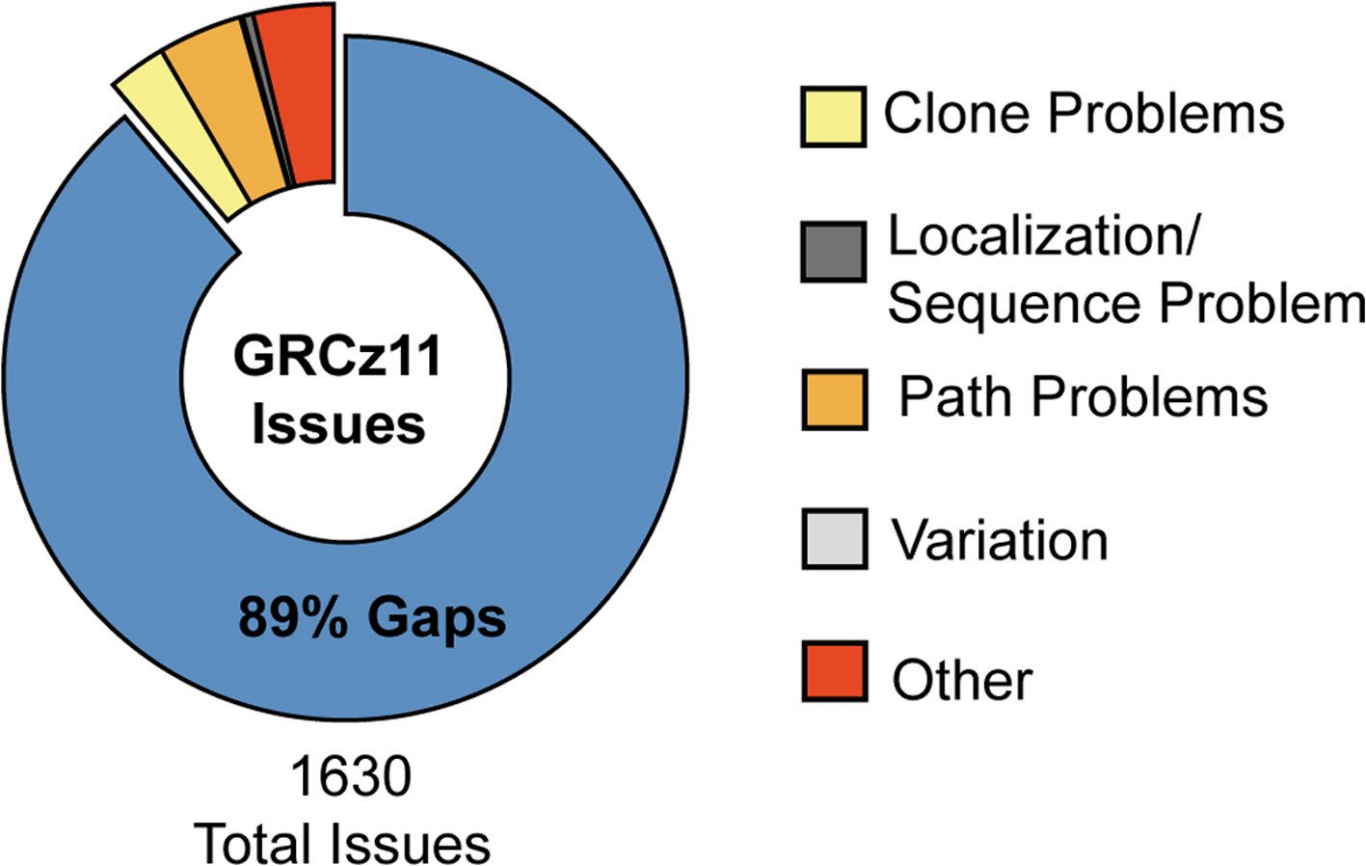
Curated current assembly issues with zebrafish reference genome GRCz11 as reported by the Genome Research Consortium

**Fig. 2:**
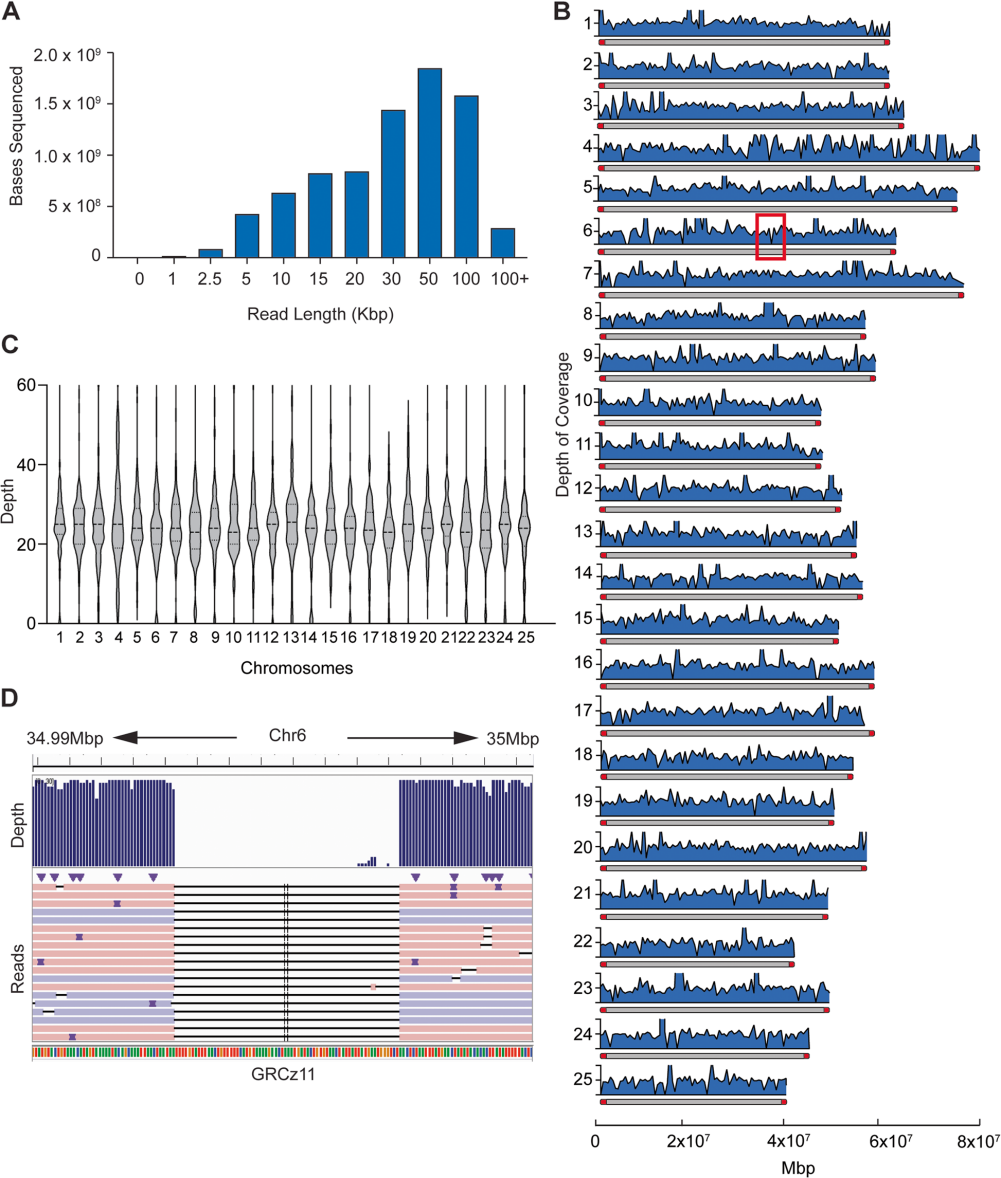
Long-read library run metrics. **A** Distribution of read lengths from one representative library (L194) relative to number of bases sequenced within that library. Read length distribution for additional libraries can be found in Supplemental Fig. 1. **B** Histogram of read depth and coverage across individual chromosomes at 50Kbp intervals. Chromosomes are depicted on the y-axis with maximum depth cut off at 50X. Telomeres (red caps) extend the first 20Kbp into each chromosome. Red box on Chr 6 emphasizes a region of low coverage. **C** Cumulative average depth across all chromosomes of long-read assembly. **D** Magnification of low coverage region depicted in **B** (red box) to show continuous nanopore reads spanning across the zero-coverage section of GRCz11

According to the Genome Research Consortium, the most significant fraction of the 1630 assembly issues within the zebrafish reference genome are gaps – ranging from a few thousand to several hundred thousand bases in length (Fig. 1). Long-read sequencing is essential for spanning these gaps and accurately mapping challenging repetitive sequences in the zebrafish genome. We tested two methods for purifying high-molecular-weight genomic DNA from a pool of muscle tissue from four mixed sex Sanger AB Tübingen (SAT) zebrafish. We used tissue from this same pool for all library preparations. We created the first library (L180) with a standard in-house DNA extraction buffer and the second (L182) using the Nanobind Tissue Big DNA Kit (Additional file 1: Fig. S1) [41]. Kit extracted DNA produced consistently longer reads (N50 = 27.8 Kbp) than the in-house method (N50 = 14.5 Kbp) and was used for all subsequent library preps (Table 1; Fig. 2A; Additional file 1: Fig. S1). Sequencing was split across six different library preparations, generating 36.9 Giga base pairs of sequence data. Although the average read length was approximately 15 Kb, most of the sequenced bases came from reads 20-150Kb in length, with the longest read spanning 464,751 base pairs (Fig. 2A).

**Table 1:** Summary of nanopore sequencing read data for *D.rerio* SAT strain

Library	Mean Read Length (bp)	N50 (bp)	Total Reads	Total Bases	Avg. Alignment
L189	7274	14,573	256,609	1.86676e+ 09	92.40%
L182	18,018	27,257	233,502	4.20726e+ 09	96.63%
L187	16,375	30,367	282,760	4.63028e+ 09	92.15%
L191	12,699	23,692	680,952	8.64755e+ 09	88.41%
L194	14,996	27,882	532,926	7.99194e+ 09	91.49%
L195	17,730	29,200	699,582	1.17051e+ 10	94.79%

The average sequencing coverage across the genome generally represents sequencing quality. However, this metric does not address the variability in coverage arising from sequencing across complex DNA templates. Sequencing reads may not adequately cover these regions; this caveat is often not factored into the average coverage across the entire genome. We examined the distribution of long reads generated across the chromosomes and assessed whether they spanned notoriously difficult to sequence regions. Generally, the long reads were evenly distributed across all chromosomes – without over or under-representing any particular region – at an average depth of ~30X (Fig. 2B-C). Next, we inspected the sequencing depth and coverage at the terminal ends of zebrafish chromosomes. Since telomeres consist of repeat regions, it is inherently challenging to align short reads to them, resulting in a loss of information and accuracy at these important genomic locations [14]. Zebrafish telomeres extend 16–20 Kbp into the chromosomes [2, 25]. Read depth at telomeres was slightly less than observed for the whole chromosomes, 24.3X for the left and 28.9X for the right telomere, respectively, but more than sufficient for long-read genome assembly (Additional file 1: Fig. S2). A reduced number of sampling points between telomeres and intrachromosomal regions likely led to the difference in sequencing depth at these locations.

Occasionally, we encountered areas of low sequence depth that justified further investigation. One such representative region exists at 35 Mbp on Chr 6 (Fig. 2B, box on Chr 6). Closer inspection of nanopore sequencing aligned to Chr 6 in the reference genome showed that all nanopore reads in that region were missing the same 70 bp sequence present in GRCz11. However, in every instance, continuous nanopore sequencing reads aligned accurately on each side of the missing 70 bp (Fig. 2D), which we believe suggests an error in the original placement of that sequence in the GRCz11, and not an issue with the long-read assembly.

### Pipeline optimization for long-read genome assembly

To assemble the zebrafish genome de novo*,* we compared two assembler tools previously used to assemble large vertebrate genomes [21, 23]. Canu, developed initially for Pacbio, is an all-in-one package that overlaps, error-corrects, and assembles long, noisy reads into contigs [21]. On the other hand, Miniasm requires a separate preceding overlap step and lacks built-in error correction but has an extremely short processing time. This latter feature is an important factor to consider when dealing with large eukaryotic genomes [23]. In addition, since nanopore sequencing is only ~ 90% accurate at the time of this study, we opted for a hybrid assembly, incorporating several polishing steps using Illumina-generated paired-end reads [26]. Table 2 summarizes assembler statistics.

**Table 2:** Summary statistics using two different assemblers relative to GRCz11

Name	Polishing	Coverage	Total Contigs	Largest Contig	NG50	Total Length	Run Time (d:h:m:s)
Canu	None	90.8%	3654	10,261,938	1,359,573	1,422,706,407	42:20:42:08
Canu_RP	Racon + Pilon	91.4%	3523	10,335,318	1,383,746	1,432,333,057	43:14:47:52
Miniasm	None	88%	1118	24,326,764	3,068,968	1,396,816,903	15:31:18
Miniasm_RP	Racon + Pilon	91.6%	1118	24,721,058	3,165,400	1,417,315,502	1:01:31:41

**Fig. 3:**
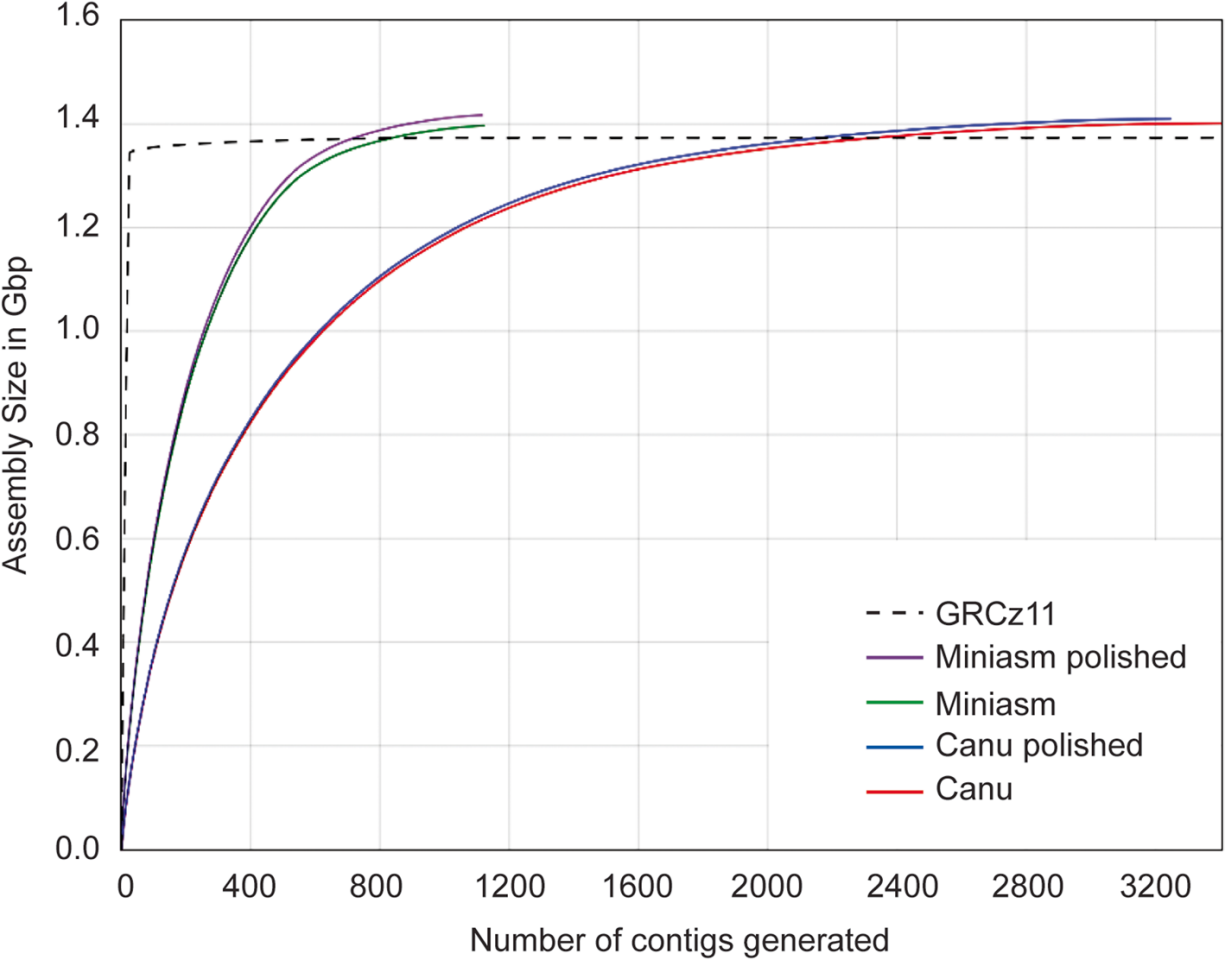
Comparison of total assembly size (Gbp) versus number of contigs generated when using Canu and Miniasm with and without polishing steps. Contigs are ordered largest to smallest, left to right

As expected of assemblers with built-in error correction, Canu generated the largest assembly (1.42 Gbp) with the highest coverage across the GRCz11 reference genome (90.8%). At the same time, Miniasm produced 1.39 Gbp of sequence at 88% coverage (Table 2). However, correcting for base-pair errors with polishing packages (Racon and Pilon) reduced the variability in length and coverage between both assemblies. Although Canu is commonly used to assemble large genomes [16, 27], we found that Miniasm surpassed it in genome coverage, contig length, and NG50 (Table 2). When comparing assembly output in terms of contig lengths and numbers, Miniasm_RP assembly covered the genome in only 1118 contigs, with the largest contig spanning an impressive 24.7 Mbp and an NG50 of 3.16 Mbp (Fig. 3 and Table 2). In addition, Miniasm required a mere day to generate the assembly while Canu processing lasted almost a month and a half. Due to overall better performance, we chose the Miniasm generated and error-corrected assembly, hereafter referred to as ZF1, for all downstream analyses.

### ZF1 assembly shows novel sequence placement

**Fig. 4:**
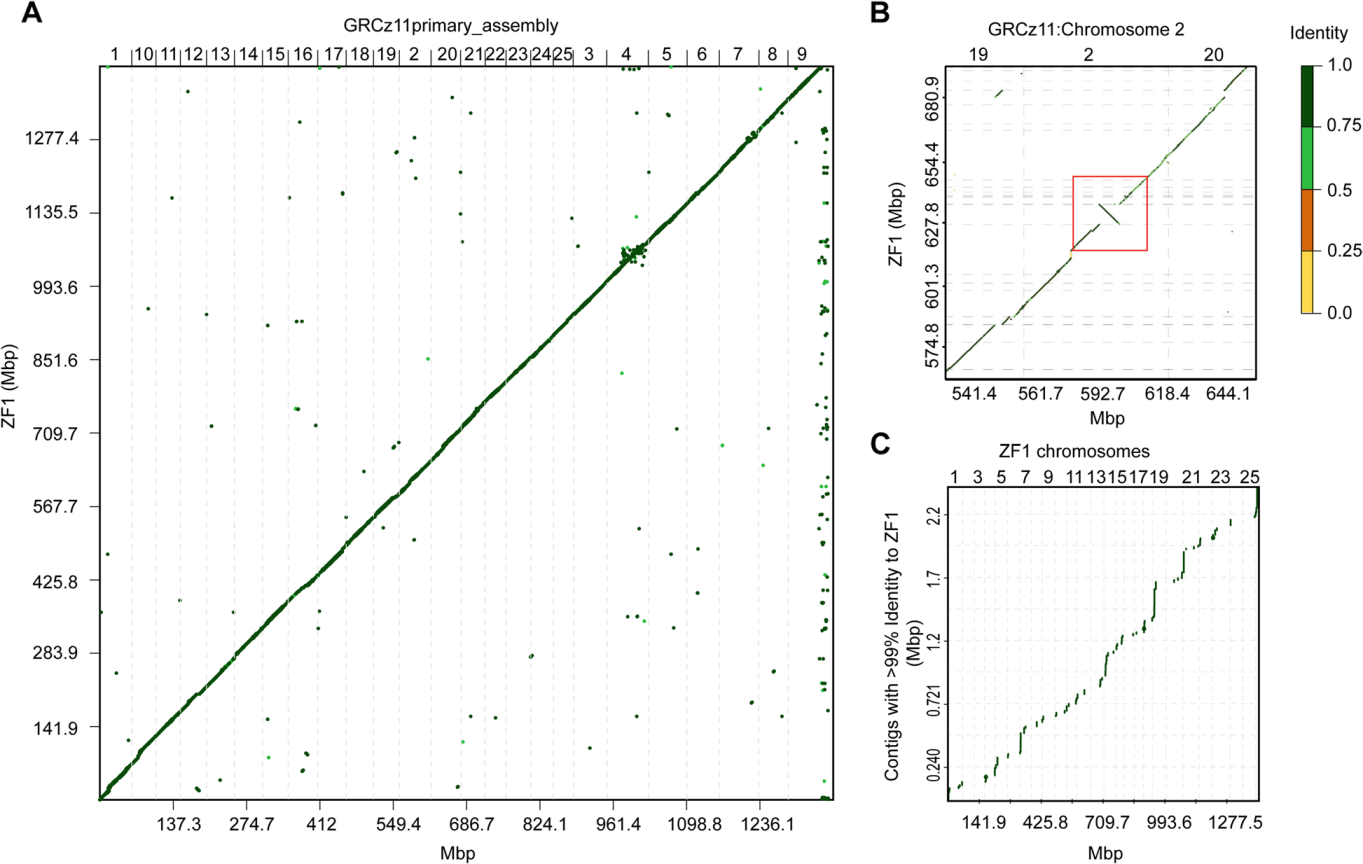
Association plots of similarities and differences between ZF1 assembly and GRCz11 primary assembly. **A** Entire de novo generated ZF1 assembly compared to GRCz11. Center, diagonal line marks strong association and alignments with shorter indels placed off-center of the diagonal (**B**) Magnified area on Chr 2 showing an 8.5Mbp inversion (red box) in ZF1 deviating from GRCz11. **C** Chromosomal placement of unlocalized contigs of GRCz11 bearing at least 99% similarity to ZF1. Color scale indicates percent similarity between alignments

To assess the accuracy of our assembler pipeline, we generated an association plot that diagrammatically depicts the alignment of our de novo assembly, ZF1, and the zebrafish reference genome assembly, GRCz11. A solid diagonal line between the two axes of the plot indicates a strong association between assemblies. A comparison between our generated assembly and the reference genome showed a solid green line of contigs from ZF1 aligned to GRCz11 (Fig. 4A), indicating our assembler pipeline was successful. However, there were crucial differences and variations between the long-read assembly and the reference genome, as indicated by small segments of alignment falling away from the diagonal line. For example, we identified a multitude of translocations and one large, 8.5 Mbp inversion residing on Chr 2 (Fig. 4B). This inversion covers over 14% of Chr 2, contains 440 protein-coding transcripts, and is large enough to span topologically associated domain (TAD) boundaries [30, 33]. Chr 4 in ZF1 contained many small (< 1 Mbp) translocations compared to GCRz11 (Additional file 1: Fig. S3). The reference sequence for Chr 4 is gene-poor and contains significant gaps, making it one of the most poorly resolved zebrafish chromosomes. A similar pattern in translocation was reported by Yang et al., when they utilized long-read sequencing to map the *D.rerio* Chr 4 [45]. The completeness of ZF1 was assessed by Benchmarking Universal Single-Copy Orthologs (BUSCO) analysis using vertebrate-specific single-copy orthologs [32]. Overall, 96.6 and 0.9% of 3354 BUSCOs were complete and partially assembled, respectively, with only 1.3% duplicated (Additional file 1: Fig. S4). Cumulatively, the analyses support the validity and accuracy of our long-read assembly.

GRCz11 contains 967 unlocalized scaffolds, or sequences not localized to a position on any specific chromosome. Cumulatively, unlocalized scaffolds make up a total of 28.3 Mbp of unplaced genomic sequence in the zebrafish genome. Since our genome-to-genome association plot showed many small alignments off the diagonal, we reasoned some of those could be newly placed unlocalized contigs from GRCz11. We filtered out scaffolds in ZF1 with less than 99% coverage since scaffolds with coverage lower than 99% could be only partially placed in the new assembly. Placement of remaining unlocalized scaffolds showed that 106 had novel locations dispersed across all chromosomes of ZF1 assembly (Fig. 4C and Additional File 1: Table S1). The remaining 861 unlocalized GRCz11 scaffolds suffered from low coverage in ZF1 at their junction points with the rest of the genome. Increasing nanopore sequencing depth would likely resolve this issue and allow these scaffolds to be assigned a chromosomal location.

### Novel Chromosomal Indels in ZF1 contain LTR Transposons

**Fig. 5:**
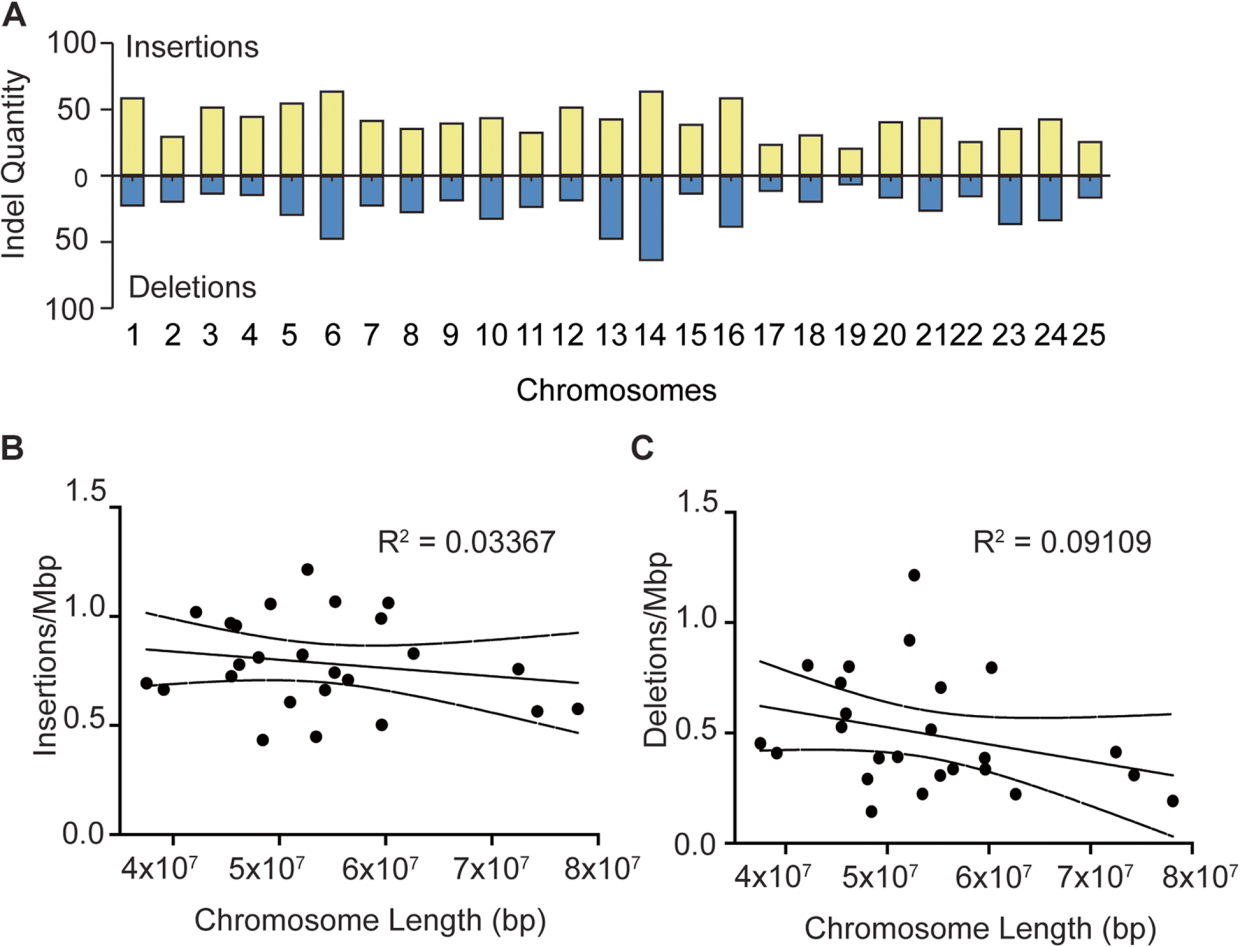
Novel indel distribution in ZF1 assembly. **A** Frequency of insertions (yellow) and deletions (blue) identified in ZF1 assembly across all chromosomes. **B-C** Dot plots showing lack of correlation between indel frequency and chromosome length. R value cutoff for correlation was set to 0.6

Next, we identified and curated the total novel insertions and deletions within the ZF1 assembly. Since genetic samples used for our assembly construction were a pool of four individuals, it was not possible for us to discriminate assembly differences from natural variation regarding SNPs and small sequence elements. Additionally, at the time of this study, nanopore-based sequencing had a base-calling error rate of approximately 10%, further reducing the accuracy of small feature identification. Therefore, we set a 1000 bp threshold for all novel genomic elements identified in ZF1 since insertions or deletions (indels) of that size are unlikely to be caused by assembly mistakes generated from base-calling errors or individual SNP variation [42]. In total, we identified 1049 insertions and 648 deletions of greater than 1000 bp across the entire zebrafish genome (Fig. 5A). We found no correlation between indel frequency and chromosome size, suggesting that indels did not randomly increase in number with increasing chromosome length (Fig. 5B-C). Instead, indel frequency is probably a factor of sequence complexity since chromosomes harboring more repeat elements are more likely to have assembly issues. To determine if any deletions in ZF1 stemmed from the mislocalized genomic sequence in GRCz11, we cross-referenced the deletions to the insertions with a minimum cutoff of 98% identity and 98% coverage. This assessment revealed that 93% (*n* = 603) of the original deletions identified in ZF1 had novel placements in other parts of the assembly (Additional file 1: Table S2).

**Fig. 6:**
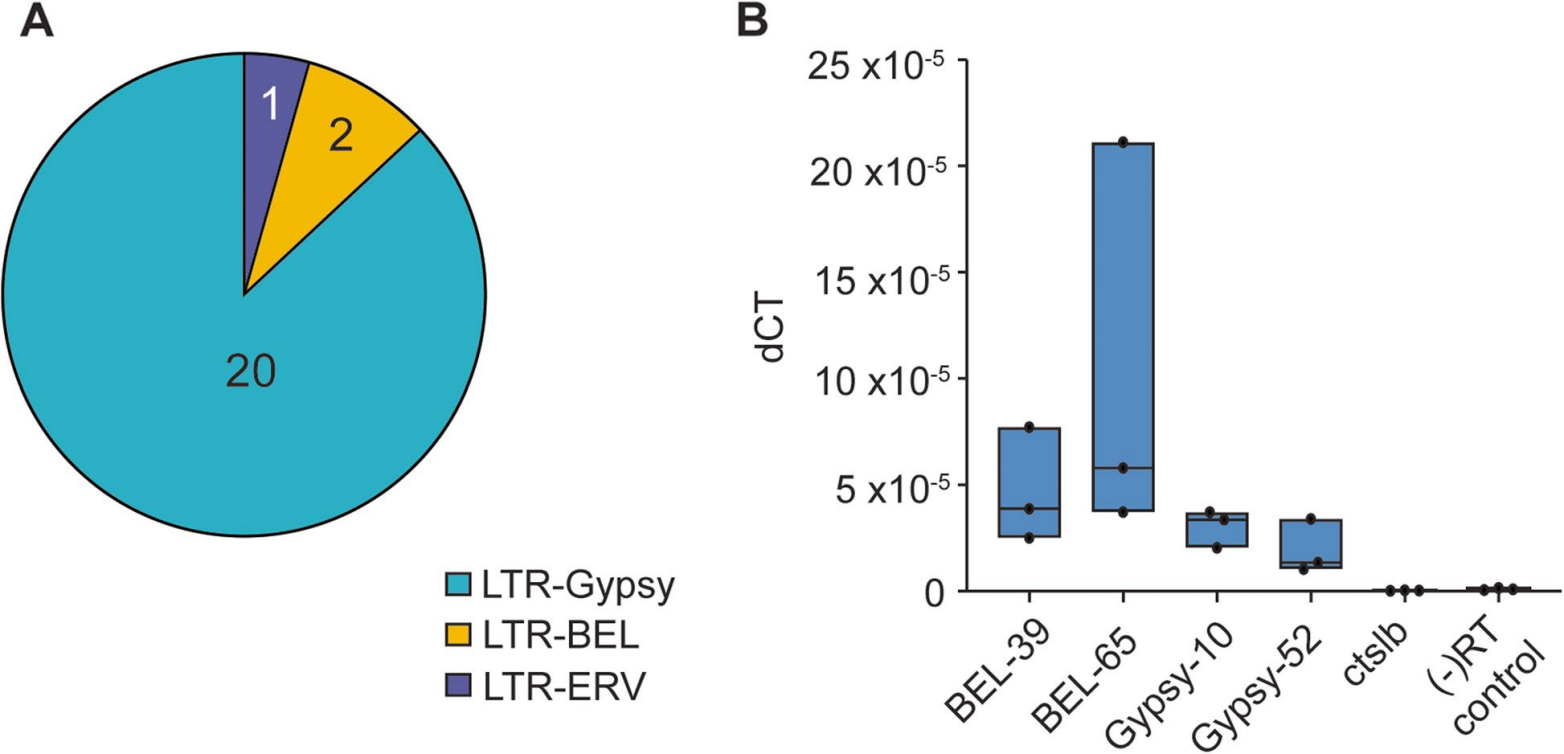
Identification of active retrotransposons in ZF1 assembly. **A** Results of gene prediction software reveals 23 novel insertions of LTR retrotransposons in the de novo assembly. **B** Expression by RT-qPCR of select retrotransposons compared to *ctslb*, which is silenced, and a negative control amplified with primers meant to pick up genomic DNA contamination

Insertions greater than 1000 bp are large enough to contain genetic elements whose regulation is likely dictated by their genomic location. We mined the 1049 insertions with gene prediction software to locate potential new genes. Geneid detected 23 protein-coding genes, all belonging to the LTR Retrotransposon family (Fig. 6A). Since repetitive elements are often difficult to map, we expected most of these LTR retrotransposons to be present in the deletion dataset, indicating their original misplacement in the reference genome. We chose four representative LTR retrotransposon indels from the 23 candidates to interrogate their original genomic coordinates in GRCz11. Considering that specific LTR retrotransposons can occur numerous times in the genome, we investigated every occurrence as a potential source of the indel. To reduce the chance of mistaking one LTR retrotransposon species for another due to high sequence similarity, we used a minimum identity cutoff of 99%. To obtain their original location, we BLASTed the four indel LTR retrotransposons identified in ZF1 against GRCz11. We then compared that region against ZF1 to detect the presence of the LTR retrotransposon of interest. Three of the four LTR retrotransposons interrogated retained all their genomic locations from GRCz11 in ZF1 (Table 3), while Gypsy52-I_DR was missing in 2 of its five genomic coordinates in ZF1, possibly due to errors in the reference genome assembly. These data indicate that strain-specific differences within the zebrafish deviate from the published reference genome. Since assembly errors in GRCz11 could not explain all of the novel insertions of the interrogated transposons, we presumed their integrations might be due to activity in the genome.

**Table 3:** Locations of 4 select LTR retrotransposons in the ZF1 assembly and GRCz11

Feature Name	New Location in ZF1		Original Location in GRCz11		GRCz11 location retained on ZF1?
	contig	chrom	chrom	location	
LTR-BEL39_Dre_I	utg000287I	2	18	31,647,198–31,653,445	YES
LTR-Gypsy10-I_DR	utg000090I	12	4	65,257,910–65,260,016	YES
			4	37,351,615–37,353,721	YES
			4	37,367,146–37,369,252	YES
			4	37,359,381–37,361,486	YES
			4	61,665,311–61,667,417	YES
			23	15,492,784–15,494,890	YES
LTR-BEL65_Dre_I	utg000174l	14	22	24,483,340–24,487,564	YES
LTR:Gypsy52-I_DR	utg000174l	14	21	17,987,219–17,992,432	NO
			1	53,792,801–53,798,011	YES
			17	21,872,111–21,877,324	YES
			24	20,991,265–20,996,478	NO
			16	3,788,049–3,793,262	YES

The expression and activity of some transposable elements are necessary for the regulation of gene expression and as functional components of nuclear architecture [18, 29, 35]. LTR retrotransposon mobility depends on the presence of expressed mRNA, which is reverse-transcribed and re-inserted into new sites in the genome [43]. In this manner, their activity manifests as novel genomic integrations while retaining the placement of their original copies. To investigate LTR retrotransposon activity, we monitored the mRNA expression levels of the four indel LTRs in 3-week-old zebrafish. We compared these to the mRNA abundance of cathepsin Lb (*ctslb*), a gene that is silenced in zebrafish post-hatching (Fig. 6B). As expected, the expression level of *ctslb* was almost undetectable in adult SAT strain zebrafish. Primers designed to amplify genomic DNA in the absence of reverse transcription produced a low signal, indicating that samples have very little genomic DNA contamination. LTR expression, however, was present above that of the silenced gene or possible genomic contamination, confirming their expression in the host cell.

## Discussion

Several groups have undertaken zebrafish genome sequencing using nanopore technology (GCA_020184715.1, GCA_903684855.2, GCA_903684865.1). Although these high-quality assemblies have been deposited into the NCBI genome repository, they remain unpublished, and the methodology by which they were built is unknown. Previous challenges in assembling the zebrafish genome stemmed from the technological limitations of short-read sequencing and the complexity of deciphering the throng of repetitive elements that comprise more than 50% of the entire zebrafish genomic landscape [7]. Additionally, an overabundance of repetitive sequence can contribute to PCR artifacts during library preparation, further confounding mapping and assembly. Thus, we set out to resolve the current reference genome issues plaguing GRCz11 and document the assembly pipeline by comparing two commonly used assembler packages and polishing tools.

We compared four different assembly pipelines to generate the most accurate assembly build. Although the Canu-generated assembly was slightly larger than the Miniasm assembly, Miniasm outperformed Canu in several quality metrics such as NG50, total contig number, and size. In addition, Miniasm required mere hours to complete the assembly process compared to the incredible CPU requirement of more than 40 days for Canu. In total, our ZF1 assembly added 43.86 Mbp of sequence to the zebrafish genome, equivalent to the size of an entire chromosome, and imparted chromosomal coordinates to 107 scaffolds previously unlocalized in GRCz11. We also identified a sizeable 8 Mbp inversion on Chr 2, which holds potential biological significance since its size is large enough to encompass multiple regulatory regions such as topologically associated domains, or TADs [11, 30]. TADs are structural chromosomal domains that maintain preferential intra-domain interactions and are subject to gene regulation based on their location and placement relative to other long-range enhancers. Thus, gene expression might be regulated differently based on which TAD it is associated with [33]. The 8 Mbp inversion completely reorganizes the placement of hundreds of genes, whose regulation is subject to change based on their updated genomic coordinates.

Interestingly, during the alignment of reads to the GRCz11 reference, we identified 45 regions which seemed to contain “gaps” in coverage, with all reads terminating at the same base pair (Fig. 2D). Upon closer inspection, we determined that reads on either side of the gap were continuous. The presence of continuous, well-aligned reads spanning both sides of a “low-coverage” region in the reference genome is likely explained by misassembled region within GRCz11 that did not align within the internal region of our reads. Similarly, we also investigated placement errors in the GRCz11 relative to ZF1. We identified a total of 1697 insertions and deletions greater than 1Kb. Most (608/648) deletions were also represented in the insertions group, suggesting they were misplaced in the original reference genome. Further examination of these indels identified 23 LTR retrotransposon genes present within the insertions. This finding was not surprising since transposable elements are widespread in the zebrafish genome. However, LTR retrotransposons encompass only 10% of all transposable elements in zebrafish, with DNA transposons representing 80% of the TEs [7]. The probability of randomly encountering an LTR retrotransposon within the insertions would be low relative to DNA transposons or other repeats. In addition, we found that most copies of the newly identified LTR retrotransposons were retained between GRCz11 and ZF1. These data suggest that the insertions that we found in ZF1 were not due to previously misplaced LTR retrotransposable elements. Instead, they are likely caused by non-random insertion mechanisms, such as the reverse transcription/reintegration method utilized by active retrotransposons [1, 10].

Although direct assessment of TE mobility was beyond the scope of this study, we did assess the expression of four select retrotransposons. We found them to be expressed above the level of repressed genes, suggesting they are active in the genome, at least at the transcriptional level. Transposons are active throughout critical biological and developmental processes, such as immune priming. The domestication of retrotransposons is also one mechanism by which new genes form [8, 9, 19, 39, 46]. Gypsy, for example, is documented to be mobile and infectious in *Drosophila*, actively remodeling the genomic and regulatory landscape in this organism [20, 28, 39]. Although a genome-wide assessment of transposon mobility has not been carried out for zebrafish, our data strongly suggest that retrotransposons are active in the genome of adult *Danio rerio*. Thus, gene regulation within the genome should be considered dynamic and strain-specific in light of retrotransposon contributions, which are ongoing and ever-present.

Finally, it must be noted that although the pipeline selected to generate the ZF1 assembly proved robust and straightforward, further work is required to improve the quality of this assembly. As it stands, this draft would benefit tremendously from additional sequence coverage to resolve SNPs and small sequence elements. Additional sequence coverage would also contribute to a more complete assembly into chromosomal scaffolds with gene annotations. Our analysis of the novel insertions identified in ZF1 was limited only to protein-coding genes; however, analysis of other genetic elements, such as regulatory RNAs and enhancers, can shed more light on the possible function of the aforementioned insertions.

## Conclusions

Zebrafish have emerged as a robust genetic tool for modeling human disease, although an inaccurate zebrafish reference genome assembly has plagued researchers for years. An accurate genomic assembly is necessary to make valid interspecies comparisons and link specific genetic events to disease model phenotypes. We have used long-read nanopore sequencing to resolve the issues of the current reference assembly and define a pipeline for generating such an assembly. Our new assembly identifies novel insertions and deletions and localizes previously unplaced genomic contigs. Our discovery of transposon activity also emphasizes the dynamic nature of the zebrafish genomic landscape and highlights the need for more frequent and accurate sequencing of model genomes.

## Methods

### DNA extraction and library preparation

All genomic samples were obtained using the SAT (#ZL1941) zebrafish line acquired from the Zebrafish International Resource Center (ZIRC). The SAT line is a derivative of a cross between Sanger AB and Tubingen double haploid individuals. To generate high-molecular-weight (HMW) genomic DNA a pool of 4 mixed sex SAT fish were sacrificed by tricaine (MS-222) overdose as follows. Fish were immersed in 250 mg/l pH buffered tricaine solution for 30 min followed by 1 h immersion in ice water. Cessation of life was confirmed by lack of heartbeat and opercular movement. Tail muscle tissue from all four animals was pooled and flash frozen in 25 mg aliquots. DNA extraction for the 1st library was carried out using a house-made extraction buffer (10 mM Tris pH 8.2, 10 mM EDTA, 200 mM NaCl, 0.5% SDS, and 0.2 mg/ul Proteinase K) and the Westerfield DNA extraction protocol [41]. All subsequent libraries were generated with DNA extracted using the Nanobind Tissue Big DNA Kit (Circulomics NB-900-701-01) using their Standard TissueRuptor Protocol – HMW. Following extraction, DNA was allowed to rest 24-48 h to solubilize into the solution. Solubilized DNA was size selected with SRE Short Read Eliminator Kit (Circulomics SS-100-101-01) according to manufacturer’s protocol, and 1.5 μg was used as input for library prep. Six libraries were generated using the Oxford Nanopore 1D Genomic DNA by Ligation Sequencing Kit (SQK-LSK109) according to the protocol provided except for the following optimizations for HMW DNA. The End Prep/Repair step was increased to 60 min, and the Adapter Ligation incubation was carried out for 10 h. Four hundred to six hundred nanogram of each prepared library was loaded in 75 μl volume onto flow cells and run for 24-30 h, until flow cell extinction, for an average N50 of 27.2Kb. An aliquot of the gDNA used for nanopore library prep was also sequenced using Illumina Hiseq 4000 platform at a depth of >50x by GENEWIZ.

### Assembly Pipeline

Raw fast5 data generated by the nanopore sequencer was base-called using Guppy [42], and all mapping was performed with Minimap2 (v2.16) [23]. Samtools (v1.10) [24] was used in index generating, alignment file sorting, and alignment statistics calculations. Assemblies were generated using two pipelines. We first used Canu (v1.9) [21] and the following source code: canu -d ../Canu -p ZF1 genomeSize = 1.4 g useGrid = false -nanopore-raw ../FASTQ/ZF1.fastq. The second used Minimap2 to first generate the pairwise mapping (PAF) file: minimap2 -x ava-ont -r 10,000 -t 16 ../FASTQ/ZF1.fastq ../FASTQ/ZF1.fastq> ../MINI_OUT/ZF1_overlap.paf. This was used as input for Miniasm (v0.3) to create the assembly: miniasm -f ../FASTQ/ZF1.fastq ../MINI_OUT/ZF1_overlap.paf > ../MINI_OUT/ZF1.gfa. The awk was used to write the assembly file:

awk ‘$1 ~/S/ {print “>“$2″\n”$3}’ ../MINI_OUT/ZF1.gfa > ../MINI_OUT/ZF1.fasta.

Polishing was performed in two ways. First, the pairwise mapping format files of the unpolished assembly and the raw long reads were generated using Minimap2: minimap2 -t 16 ../MINI_OUT/ZF1_MM.fasta ../FASTQ/ZF1.fastq > ../MINI_OUT/ZF1_overlap_for_polishing.paf, followed by Racon (v1.4.13) [37] to polish the unpolished assemblies using the raw long reads. Next, short-read polishing using Pilon (v1.23) [38] was performed using Illumina whole-genome sequencing data. The alignment files of raw reads to the assembly were first generated using bwa (v0.7.17) and indexed using Samtools (v1.10). Then the Pilon (v1.23) was used to polish the assembly using the short reads alignment: pilon -Xmx160G --genome. /FASTA/ZF1_MM_R_lr.fasta --fix all --changes --bam. /BAM/ZF1_MM_R_lr_sr_mapping.sorted.bam --threads 32 --output. /pilon_canu/pilon_round1 | tee. /pilon_canu/round1.pilon.

### Variant calling and genetic element identification

The paftools.js in Minimap2 (v2.16-r922) was used to call variants from the generated assembly against the reference. minimap2 -cx asm5 --cs. /ZF_Ref/Danio_rerio.GRCz11.dna.primary_assembly.fa. /Assemblies/ZF1_MM_R_lr_R_sr.fasta \ | sort -k6,6 -k8,8n \ | paftools.js call -f. /ZF_Ref/Danio_rerio.GRCz11.dna.primary_assembly.fa - >. /VCF/ZF1_MM_R_lr_R_sr.vcf. From the generated VCF file, the indels with size larger than or equal to 1000 bases were selected by checking the sequencing lengths of the ‘REF’ and ‘ALT’ column for each variant. The involved sequences were written in a FASTA file.

Genetic elements within the insertions from the VCF calling were predicted using Geneid (v1.4) [5], using the human parameter file ‘human3iso.param’ which can be used for vertebrate genomes and the following compands: geneid -XP /home/xzh289/Tools/geneid/param/human3iso.param. /1000bp_insertion/ZF1_MM_R_lr_P_sr_1000bp_insertion.fasta> ZF1_MM_R_lr_P_sr_1000bp_insertion.extend.gff. Newly discovered genetic elements were than BLASTed to confirm their identify or conserved motifs.

### Assembly completeness and accuracy

Assembly completeness was assessed with BUSCO v5.2.2 package using Vertebrata category to assess all vertebrate-specific single-copy orthologs [32]. Association dot plots comparing the ZF1 assembly to GRCz11 reference or GRCz11 unlocalized contigs bearing > 99% identity in ZF1 were carried out using the web-based version of D-Genies and .paf files previously generated by Minimap2 (v2.16-r922) [6].

### Retro-transposon locations and expression

To determine if the LTR retrotransposons identified with Geneid (v1.4) maintained their original GRCz11 genomic locations in ZF1, we mined the alignment data of ZF1 assembly to GRCz11 reference at those locations where the LTR retrotransposons of interest were shown to exist (Table 3). RNA was extracted from 3 week old SAT fish using TRIzol™ Reagent (Fisher Scientific) according to the manufacturer’s protocol to assess the expression of the four retrotransposons. All residual DNA was removed using DNA-free™ DNA Removal Kit (Life Technologies). Real-time quantitative PCR (RT-qPCR) primers (Additional file 1: Table S3) were designed to span a ~ 150 bp region of each LTR-RT. RT-qPCR was carried out for 40 cycles using iTaq Universal SYBR green Supermix according to the manufacturer’s protocol (Biorad). All gene expression was normalized to elongation factor 1-alpha (*eef1a*) housekeeping gene signal and depicted graphically as delta CT values. As a control for monitoring transcript abundance of genes that should not be expressed in adult zebrafish, we also included primers for cathepsin Lb (*ctslb*), a peptidase expressed in the hatching gland during early larval development.

## Supplementary Information

**Additional file 1: Figure S1.** Read length distribution and sequenced bases generated by each group across all libraries used in assembly generation. **Figure S2.** Tukey box and whiskers plot of average depth at the telomeric regions of all chromosomes in the zebrafish genome. **Figure S3.** Association plot of Chr 4 in ZF1 and GRCz11 assemblies illustrating many small sequence differences between the two builds. **Figure S4.** BUSCO analysis of GRCz11 reference assembly and ZF1 assembly using vertebrate-specific single-copy orthologs. **Table S1.** Chromosomal location of GRCz11 unlocalized scaffolds bearing > 99% coverage in GRCz11. **Table S2.** Deletions mapped to insertions in ZF1 assembly. **Table S3.** Primers used for RT-qPCR.

## Data Availability

The data generated in this Whole Genome Shotgun project are available at DDBJ/ENA/GenBank under the accession JAIHOL01, https://www.ncbi.nlm.nih.gov/Traces/wgs/JAIHOL01. The assembly of this sequencing data is found at https://www.ncbi.nlm.nih.gov/assembly/GCA_020064045.1/

## Abbreviations

Chr: Chromosome
Gbp: Giga base pair, 1 billion nucleotides
GRCz11: Genome Reference Consortium zebrafish reference genome assembly version 11
HMW: High molecular weight
Kbp: Kilo base pair, 1 thousand nucleotides
LTR: Long terminal repeat
Mbp: Mega base pair, 1 million bases
NGS: Next-generation sequencing, i.e., Illumina sequencing
SAT: Sanger AB Tübingen, a common lab strain of zebrafish
TAD: Topologically associated domain
TE: Transposable element
