# Supplementary material for: Long-read sequencing of the zebrafish genome reorganizes genomic architecture
Source: BMC Genomics. 2022 Feb 10;23:116. doi: 10.1186/s12864-022-08349-3 (PMC8832730; doi:10.1186/s12864-022-08349-3)
Supplement: Supplementary file 1 — Additional file 1: Figure S1. Read length distribution and sequenced bases generated by each group across all libraries used in assembly generation. Figure S2. Tukey box and whiskers plot of average depth at the telomeric regions of all chromosomes in the zebrafish genome. Figure S3. Association plot of Chr 4 in ZF1 and GRCz11 assemblies illustrating many small sequence differences between the two builds. Figure S4. BUSCO analysis of GRCz11 reference assembly and ZF1 assembly using vertebrate-specific single-copy orthologs. Table S1. Chromosomal location of GRCz11 unlocalized scaffolds bearing > 99% coverage in GRCz11. Table S2. Deletions mapped to insertions in ZF1 assembly. Table S3. Primers used for RT-qPCR. [file 12864_2022_8349_MOESM1_ESM.zip › Supplemental_Figures_v1.docx]

**Supplemental Figures**


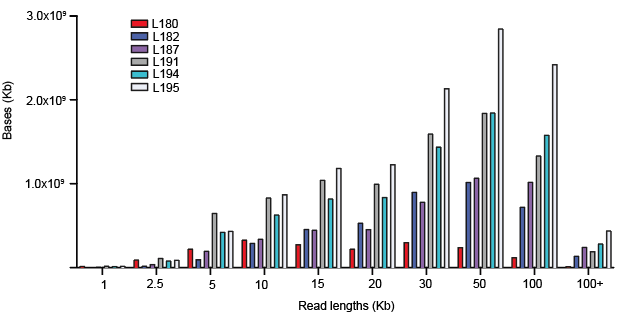


**Figure S1**. Read length distribution and sequenced bases generated by each group across all libraries used in assembly generation.


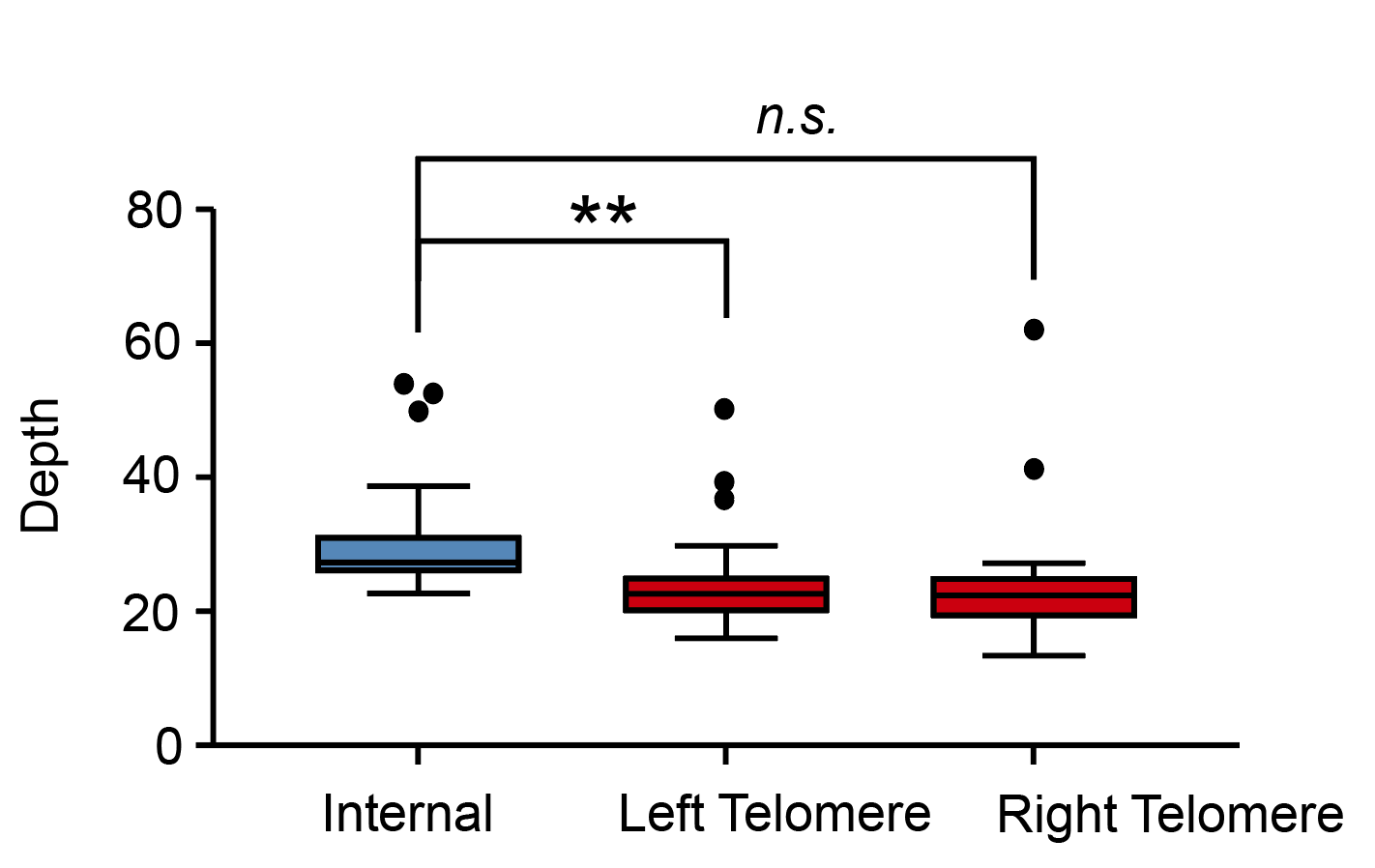


**Figure S2**. Tukey box and whiskers plot of average depth at the telomeric regions of all chromosomes in zebrafish genome. Significance between telomeric and intra-chromosomal depth was calculated by t-test, p-value 0.0097 (**), *n.s.* is no significance.


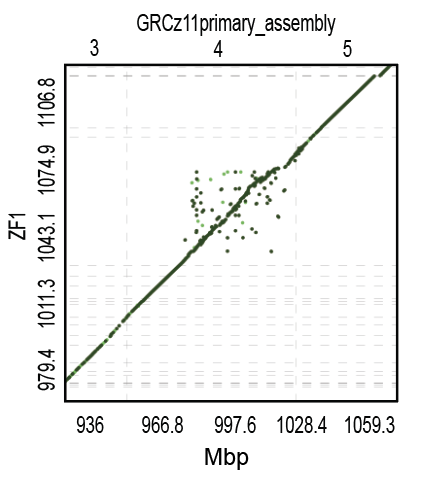


**Supp. Figure 3**. Association plot of Chr 4 in ZF1 and GRCz11 assemblies illustrating many small sequence differences between the two builds.


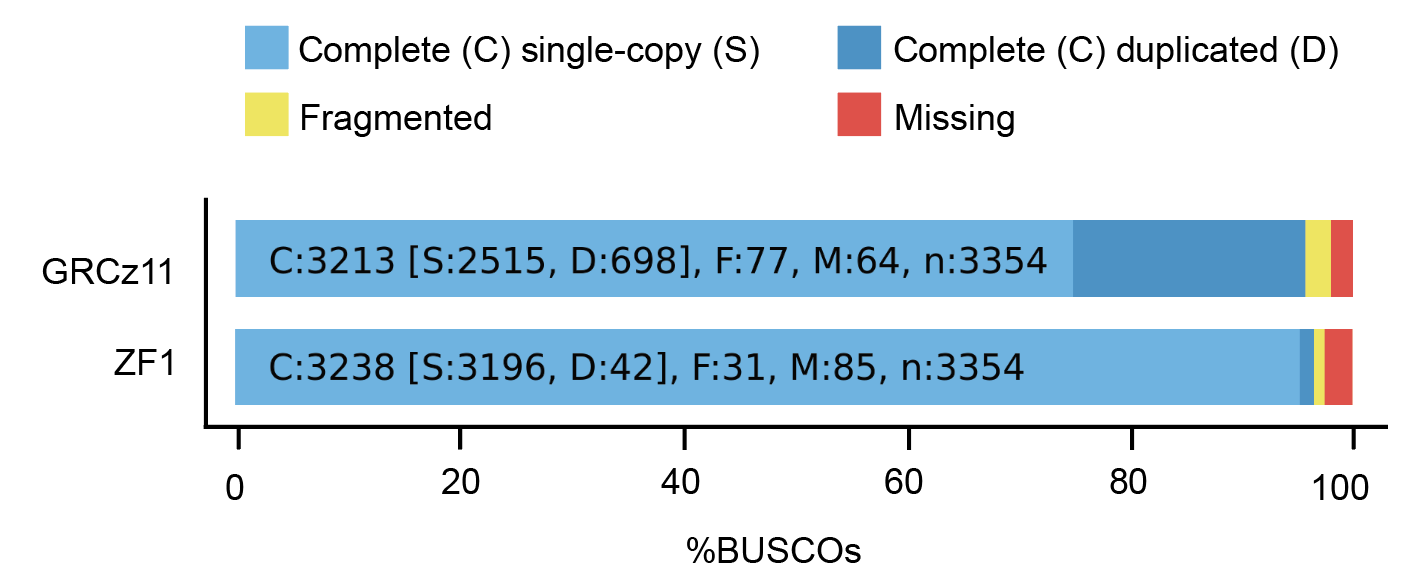


**Supp. Figure 4**. BUSCO analysis of GRCz11 reference assembly and ZF1 assembly using vertebrate-specific single-copy orthologs.
